# Evolution of epithelial morphogenesis: phenotypic integration across multiple levels of biological organization

**DOI:** 10.3389/fgene.2015.00303

**Published:** 2015-09-29

**Authors:** Thorsten Horn, Maarten Hilbrant, Kristen A. Panfilio

**Affiliations:** Institute for Developmental Biology, University of Cologne, Cologne, Germany

**Keywords:** epithelial morphogenesis, evolution of development, insects, extraembryonic tissues, *Hox3/zen*, *Tribolium castaneum*, *Megaselia abdita*, *Oncopeltus fasciatus*

## Abstract

Morphogenesis involves the dynamic reorganization of cell and tissue shapes to create the three-dimensional body. Intriguingly, different species have evolved different morphogenetic processes to achieve the same general outcomes during embryonic development. How are meaningful comparisons between species made, and where do the differences lie? In this Perspective, we argue that examining the evolution of embryonic morphogenesis requires the simultaneous consideration of different levels of biological organization: (1) genes, (2) cells, (3) tissues, and (4) the entire egg, or other gestational context. To illustrate the importance of integrating these levels, we use the extraembryonic epithelia of insects—a lineage-specific innovation and evolutionary hotspot—as an exemplary case study. We discuss how recent functional data, primarily from RNAi experiments targeting the Hox3/Zen and U-shaped group transcription factors, provide insights into developmental processes at all four levels. Comparisons of these data from several species both challenge and inform our understanding of homology, in assessing how the process of epithelial morphogenesis has itself evolved.

## Introduction

In the rapidly developing fruit fly *Drosophila melanogaster*, the predominant insect model for developmental genetics, embryonic morphogenesis occurs largely after cell fates are determined. Indeed, there is extensive literature on *Drosophila* early tissue patterning, including axis specification and segmentation, preceding morphogenesis. Perhaps as a result of our profound knowledge in *Drosophila*, many evolutionary developmental (evo-devo) studies in arthropods take a gene-centered approach and focus on early patterning, as early fate specification is often a powerful signal for comparisons of species that are separated by long periods of evolutionary time (e.g., Peel et al., 2005; Sachs et al., 2015).

In this Perspective article, however, we highlight the importance of studying the morphogenetic movements that occur during animal development and of integrating multiple levels of biological organization when making interspecific comparisons. For doing so, we distinguish between four increasingly inclusive levels of biological organization. (1) Genetic regulation of development comprises information about the specific genes and their protein products that are involved in transcriptional control, signaling cascades, and the molecular basis of cytoskeletal structure and remodeling. (2) Individual cells differentiate to acquire a particular identity, including the transcriptional state as well as cell shape and structure. (3) More broadly, cells coordinate with their neighbors within tissues. In epithelial tissues for example, cells retain contact with their neighbors via adherens junctions, such that cell shape changes affect the entire tissue’s geometry. (4) Finally, the egg is a global system, where tissue integrity and inter-tissue adhesion need to be precisely controlled during morphogenesis to achieve the final form.

Overall, integration of different biological levels is as much a conceptual framework for understanding the physical context of a given gene’s role in a developmental process as for interpreting how morphogenesis has evolved. To illustrate this, here we discuss recent advances in the study of extraembryonic (EE) development in a range of insect model species. We show that the insect EE epithelia provide a case study with a particularly rich evolutionary history, making them well suited to assessing the evolution of morphogenesis.

## Development and Evolution of the Insect Extraembryonic Membranes

**FIGURE 1:**
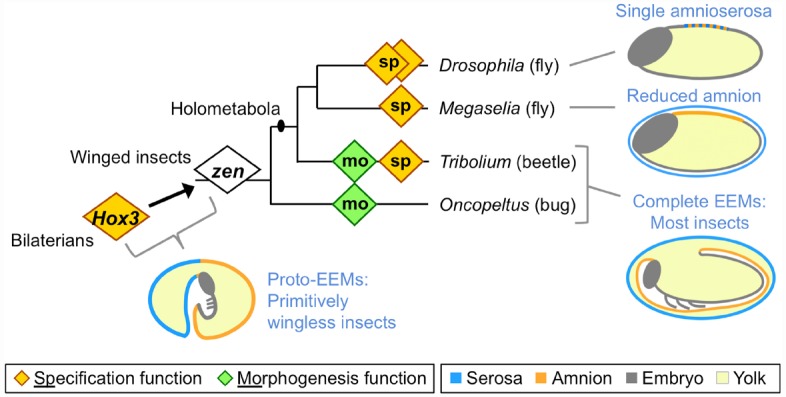
**Evolution of extraembryonic membranes (EEMs) and ***zen*** gene function in insects.** This phylogeny shows species for which functional data on the homeodomain transcription factor Zen are available, and which are discussed here. Diamonds represent individual *zen* genes (two each in *Drosophila* and *Tribolium*), with either a late morphogenetic function (green) or an early specification function (orange). Non-insect *Hox3* orthologs also have a specification function, albeit within embryonic rather than extraembryonic tissue. Note that within the fly lineage the highly divergent *bicoid* paralog has been omitted for clarity (for recent work on this, see Klomp et al., 2015). Schematics show evolutionary stages of EEM acquisition and secondary reduction as inferred from extant species (blue text; color coding is indicated in the legend). Here, “complete” refers to the formation of discrete, closed compartments within the egg, namely the outer serosal sac and the inner amniotic cavity. The illustration of EEM organization in primitively wingless insects is modified from (Panfilio, 2008), with the corresponding author’s consent.

Many arthropod eggs include an EE tissue component, but in the insects this feature has become a specific structural innovation (reviewed in Panfilio, 2008). At the base of the winged insect lineage, the EE epithelial membranes evolved to form discrete compartments within the egg. In most winged insects, the serosa lines the eggshell, providing the outermost cellular layer and enclosing all other contents, including the yolk. The amnion, analogous to its namesake in vertebrates, forms a fluid-filled cavity ventral to the embryo, retaining a connection to the embryo along the latter’s dorsal margin (Figure 1: “most insects” schematic).

The ability of EE membranes to form these compartments early in development has allowed the insects to exploit diverse ecological niches, largely due to the manifold functions of the serosa as a protective outer layer that buffers the embryo against environmental fluctuations and assaults. Recent work has shown that serosal cuticle secretion correlates with the acquisition of desiccation resistance, and that the cuticle itself provides mechanical support to the egg (Rezende et al., 2008; Jacobs et al., 2013; Panfilio et al., 2013). At the same time, recent experimental evidence demonstrates the long hypothesized ability of the serosa to protect the embryo after wounding and pathogen infection via upregulation of the innate immune system (Chen et al., 2000; Jacobs et al., 2014). Furthermore, the serosa’s ultrastructure is consistent with physiological roles in water and solute processing, and it has acquired additional mechanical and physiological functions during hatching and early larval life in species with oviposition sites within plant and animal tissues (citations in Panfilio, 2008).

The functional importance of the amnion remains far more enigmatic, despite early recognition of the potential value of an insect amniotic cavity (Zeh et al., 1989). Indeed, the amniotic cavity has been lost independently during the evolution of apocritan wasps and cyclorrhaphan flies (Fleig and Sander, 1988; Rafiqi et al., 2008), with the amniotic epithelium confined to a dorsal yolk cover (Figure 1: schematic for *Megaselia*). More extremely, in *Drosophila melanogaster* the serosa and amnion are conflated into a single, dorsal amnioserosa, dispensing with EE compartments entirely (Figure 1: schematic for *Drosophila*), and some *Drosophila* species have decanalized development to the point where amnioserosal formation is variable, but still essential (Gavin-Smyth et al., 2013; Panfilio and Roth, 2013).

Using the evolution and development of insect EE membranes as a case study, in the next sections we discuss how the different levels of biological organization are interconnected. We show that, for example, changes at the gene level can induce dramatic changes in cell and tissue behavior that differ between species, even if the consequences at the whole egg level are similar. On the other hand, similar morphogenetic movements can be achieved by quite different mechanisms on the cellular and tissue organizational levels.

## Linking *zen* and U-shaped Genes to Changes at the Egg Level

Extraembryonic tissue evolution is tightly linked to evolution of the Hox3/Zen transcription factor (Figure 1). The evolutionary origin of strictly EE expression of this gene coincides with the origin of complete EE compartments (Hughes et al., 2004; Panfilio et al., 2006). Hox genes are generally highly conserved in relative genomic position, protein sequence, copy number, and function in anterior-posterior patterning (Krumlauf, 1992; Cook et al., 2001). In contrast, arthropod *Hox3* orthologs are prone to duplication and marked sequence divergence, particularly the insect orthologs (known as “*zen*,” after the original *Drosophila* mutants), with independent instances of duplication in beetles, flies, and lepidopterans (Pultz et al., 1988; Brown et al., 2002; Panfilio et al., 2006; Panfilio and Akam, 2007; Chai et al., 2008; Rafiqi, 2008; Ferguson et al., 2014). Interestingly, in the red flour beetle, *Tribolium castaneum*, the two *zen* paralogs have different functions (van der Zee et al., 2005), and these will be discussed in turn.

In all holometabolous insects studied so far *zen*, or *Tc-zen1* in *Tribolium*, has a conserved function in EE tissue specification (Figure 1: orange diamonds). However, while loss of function mutation in *Drosophila* is lethal (Wakimoto et al., 1984), the scuttle fly *Megaselia abdita* and *Tribolium* can survive after RNA interference (RNAi) knockdown (van der Zee et al., 2005; Rafiqi et al., 2008; Panfilio et al., 2013). Examining why the end-stage phenotypes differ after loss of a conserved gene’s function provides a good example for the integration of the different levels of biological organization.

Key to understanding the different phenotypic outcomes of disrupting *zen/zen1* is the evolutionary change in EE membrane complement between these species. The single amnioserosa of *Drosophila* exhibits features of both early serosa and late amnion (reviewed in Schmidt-Ott et al., 2010), and specification of the entire EE domain is under the control of *Dm-zen* (the paralog *Dm-z2* is not essential during embryogenesis, Pultz et al., 1988). In the less derived situation in *Megaselia* and *Tribolium*, *Ma-zen*/*Tc-zen1* only specifies the serosa (van der Zee et al., 2005; Rafiqi et al., 2008). The phenotypic outcome after *zen* knockdown could then be explained by loss of all EE tissue identity in *Drosophila*, while *Megaselia* and *Tribolium* retain an amnion.

However, on closer inspection the similarity in gene function, residual EE tissue complement, and end-stage phenotypic outcome in *Megaselia* and *Tribolium* is rather surprising if we consider the difference in EE membrane configuration (Figure 1: schematics). In both species, we observe a respecification from serosal to amniotic fate and in both species it is important to have a tissue covering the yolk dorsally during the dorsal closure stage. However, the underlying wild type configurations are different. In *Megaselia*, the amnion provides a persistent dorsal yolk cover, and its overall shape, size, and dorsal position are not changed dramatically by the *Ma-zen^RNAi^* fate shift (Rafiqi et al., 2008). In contrast, in *Tribolium* the dorsal side of the egg is first covered by the serosa and only later in wild type development is the amnion pulled dorsally when the serosa contracts (Panfilio et al., 2013). How can *Tribolium* then survive without a serosa? Here, *Tc-zen1^RNAi^* not only produces a persistently dorsal amniotic region due to respecification (van der Zee et al., 2005), but also reveals novel cellular and tissue properties of the entire amnion in late development as it takes over the role of the serosa in providing a dorsal cover (Panfilio et al., 2013). Hence, the survival of *Ma-zen^RNAi^* embryos is rather due to the dispensability of the serosa for dorsal closure, while in *Tribolium* developmental regulation—that is, compensation via plasticity of the amnion—enables survival after *Tc-zen1^RNAi^*. Thus, conserved, early gene functions can feed into different developmental routes, depending on tissue configuration and morphogenetic properties.

At the same time, other genes with EE roles have undergone changes in their particular function and in their interaction partners during insect evolution. One example is the T-box transcription factor Dorsocross (Doc), a member of the U-shaped gene family (Frank and Rushlow, 1996; Reim et al., 2003). In *Drosophila*, *Dm-Doc* is necessary for the maintenance of the amnioserosa toward the end of germband extension, when Zen protein disappears (Reim et al., 2003, and references therein). In contrast, *Tc-Doc* has multiple roles in *Tribolium* EE morphogenesis, but no role in maintaining either EE tissue (TH, KAP unpublished observation). There is some evidence that *Ma-Doc* has a maintenance function in the *Megaselia* serosa (Rafiqi et al., 2008), but the end stage RNAi phenotype would also be consistent with an early morphogenetic role, as in *Tribolium*.

Consistent with this difference in the EE role of Doc, the molecular context of its function also differs between species. *Drosophila Doc* expression requires simultaneous inputs from Dm-Zen and Dm-Dpp (Reim et al., 2003). In contrast, in *Tribolium* these inputs are temporally and spatially distinct, and subsequent Dpp signaling is itself locally dependent on *Tc-Doc* (TH, KAP unpublished observation), a feature not known from *Drosophila*. Another example is *Doc*’s relation to *hindsight* (*hnt*), another U-shaped gene. In *Drosophila*, *Dm-hnt* is downstream of *Dm-Doc* and therefore shows a similar knockdown phenotype. In *Tribolium*, both genes also show a similar knockdown phenotype to one another, but they seem not to influence each other’s expression (TH, KAP unpublished observation).

Finally, *Dm-Doc* performs multiple functions within the body proper (Hamaguchi et al., 2012; Sui et al., 2012), such as for heart development (Reim and Frasch, 2005), that are not observed in *Tribolium* (Nunes da Fonseca et al., 2010). Interestingly, one of these functions, bending of the *Drosophila* wing imaginal disc, directly links the transcription factor Dm-Doc to cellular and epithelial rearrangements (Sui et al., 2012). Here, Dm-Doc promotes intracellular microtubule web redistribution and degradation of the extracellular matrix through Matrix metalloproteinase. It remains to be seen if similar mechanisms are also employed downstream of Doc in EE morphogenesis across species.

In summary, disruption of *zen*, a gene with a conserved function in specification of the serosa, leads to lethality, compensation by the amnion, or simply loss of the serosa with no severe consequences for development, depending on the tissue topography of the species under investigation. Moreover, there are large differences in gene knockdown phenotypes, overall gene functions, and specific interaction partners between orthologous genes in different species, and these differences can only be understood if all other biological levels, from cells to the egg system, are taken into account.

In the next section we shift the focus from the genes themselves to tissue organization and function, again highlighting differences between species at different levels of biological organization.

## Linking Cellular, Tissue, and Egg System Levels

In late embryogenesis, it is essential that insect EE tissue actively withdraws in a precise way to mediate dorsal closure, whereby the embryonic epidermis seals at the dorsal midline and EE tissue degenerates within the yolk. Indeed, amnioserosa-epidermal tissue coordination during *Drosophila* dorsal closure has been extensively studied over the last 15 years (e.g., Jacinto et al., 2000; Kiehart et al., 2000; Solon et al., 2009; Lada et al., 2012; Wells et al., 2014). While differences in tissue organization are expected between dorsal closure involving an amnioserosa and dorsal closure involving a serosa and amnion, we also find differences between species with both EE membranes (Figure 1: “most insects” schematic). To illustrate this point, here we compare late EE morphogenesis between *Tribolium* and the hemimetabolous milkweed bug, *Oncopeltus fasciatus*, charting a sequence of similarities and differences as morphogenesis proceeds.

Firstly, rupture of the EE tissues over the embryo’s head produces an opening through which the embryo passively emerges. In *Oncopeltus*, preparation for EE rupture within this specialized region involves apoptosis of the amniotic cells subjacent to the serosa, thinning the region to a single EE epithelium, while at the border of this region the amnion adheres strongly to the serosa (Panfilio and Roth, 2010). As this epithelial remodeling occurs locally, the entire egg system is subtly reorganized to ensure that the specialized EE region is centered at the egg pole, which appears to mechanically facilitate rupture via global contractile force exerted by the serosa (Panfilio, 2009; Panfilio and Roth, 2010). In contrast, in *Tribolium* the opening for EE rupture is not centered at the egg pole but occurs anterior-ventrally (Panfilio et al., 2013). Here, precision in determining the site of EE opening involves morphological specialization in a cap of amniotic cells. Furthermore, preparation for rupture in *Tribolium* involves the formation of an amnion-serosa epithelial bilayer over most of the amnion’s surface area (Koelzer et al., 2015), not just the narrow ring of amnion-serosa contact seen in *Oncopeltus*. These differences in local behavior of the amnion and in the amnion-serosa connection are all the more striking given that *Of-zen* and *Tc-zen2*, the second *Tribolium* paralog, both act extraembryonically to ensure that EE rupture occurs (Figure 1: green diamonds; van der Zee et al., 2005; Panfilio et al., 2006).

**FIGURE 2:**
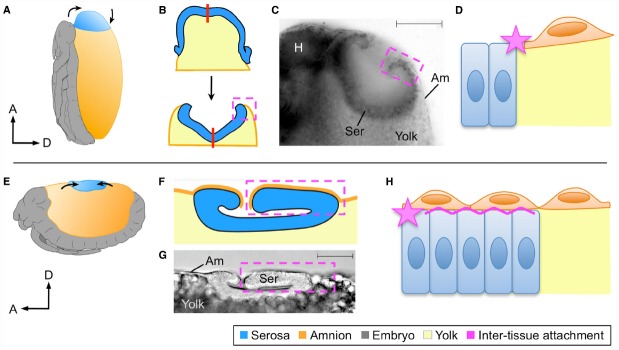
**Different tissue organizations achieve the same morphogenetic outcomes.** At the dorsal organ stage the serosa compacts into a hollow disc that sinks into the yolk, shown here for *Oncopeltus fasciatus* **(A–D)** and *Tribolium castaneum* **(E–H)** as representative of hemi- and holometabolous insects, respectively. Although the site of dorsal organ formation differs in relative position and geometry within the global egg system **(A,E)**, in both cases serosal cells become columnar as the tissue everts **(B,C,F,G)**. The process of serosal eversion is shown schematically in B, where the red line indicates the center of the serosa during this process. In yet another difference, the nature of amnion-serosa attachment consists of a lateral junction within the plane of the extraembryonic epithelium in *Oncopeltus* (D: star), while the bilayered arrangement in *Tribolium* additionally involves basal–basal contact of the two tissues (H: zigzag line). All views are lateral except **(B)**, which is dorsal and omits the embryonic tissue for simplification. Dashed boxes indicate the region of inter-tissue attachment, which is shown schematically at the cellular level in **(D)** and **(H)**. Micrographs show fixed embryos with a nuclear stain **(C)** or DIC illumination **(D)**, with scale bars of 100 μm and 50 μm, respectively. Abbreviations: A, anterior; Am, amnion; D, dorsal; H, head; Ser, serosa. Images A,B,C,E are reproduced with minor modification from (Panfilio, 2008, 2009; Panfilio and Roth, 2010; Panfilio et al., 2013), with the corresponding author’s consent.

In subsequent stages the EE tissues withdraw dorsally, but with the serosa ending up in the tapered dorsal-anterior in *Oncopeltus* compared to the flat dorsal-medial region in *Tribolium* (Figures 2A,E). Nonetheless, in both cases the serosa transforms from a squamous to a columnar epithelium and forms a hollow disc known as the dorsal organ (Panfilio, 2009; Panfilio and Roth, 2010; Panfilio et al., 2013; Figures 2B,C,F,G). Thus, cell shape and intra-tissue organization are conserved despite the geometrical difference resulting from the tissues’ positions within an anisotropic egg system.

However, as a consequence of the manner in which the amnion-serosa connection was prepared for rupture, the inter-tissue organization remains fundamentally different at the dorsal organ stage. The *Oncopeltus* amnion is only connected to the serosa at its margin, and sits on top of the yolk (Figure 2D). While both this attachment point and substrate also apply to the *Tribolium* amnion, the bilayer organization means that additionally a portion of the amnion has the serosa as a substrate (Figure 2H). As the serosa degenerates, tissue continuity over the yolk surface is essential for successful dorsal closure (Panfilio et al., 2013). The planar (lateral–lateral) nature of amnion-serosa attachment in *Oncopeltus* allows the serosa to efficiently pinch off and draw the edges of the amnion together above it (Figure 2B). In *Tribolium*, inter-tissue shearing is required so that the portion of the amnion over the serosa (apical-basal connection) can detach, enabling final serosal internalization (Koelzer et al., 2015).

Altogether, *zen*-mediated rupture, EE contraction and withdrawal, and the cellular structure of the serosal dorsal organ are shared between *Oncopeltus* and *Tribolium* even though the manner of amniotic regionalization (selective apoptosis or morphological alteration) and therefore the nature of the amnion-serosa inter-tissue connection differ.

## Conclusions

In this Perspective, we use morphogenesis of the insect EE epithelia to show how different levels of biological organization can provide apparently contradictory signals as to the degree of evolutionary conservation across species. At first glance, these levels are hierarchically ordered, with increasing complexity toward the whole egg system: genes specify cell types and shapes, cells of similar type form tissues, and different tissues shape the whole egg system morphology. However, any pattern of congruence across levels is possible. For example, *zen* orthologs are necessary to specify the serosa in holometabolous insects, but the loss of *zen* function is lethal in some species, while others survive—variously due to serosal dispensability or morphogenetic compensation by the amnion. These phenotypic outcomes can be explained by a conserved gene function being embedded in the context of differences in EE tissue complement and topographical configuration across species. In the case of EE epithelial withdrawal in *Oncopeltus* and *Tribolium*, the nature of tissue regionalization and inter-tissue attachment differ dramatically even while gene function, intra-tissue structure, and gross morphogenesis are similar. Only the integration of all biological levels can provide the full picture and give insight into the evolution not just of epithelial morphogenesis but of embryogenesis in general, which ultimately depends on cell shape changes and coordinated tissue reorganization.

In the past, these levels have predominantly been studied separately or in limited combinations. In the watershed Heidelberg screen of *Drosophila* embryonic patterning mutants (Nüsslein-Volhard and Wieschaus, 1980), gene function was linked to final phenotype as determined from larval cuticle preparations, a method still widely employed, especially for large scale screening (Schmitt-Engel et al., 2015). However, even as the initial link between gene and egg system levels is being established, the aim is to refine this information to more precise phenotypic analysis. At this point, a misexpressed gene itself becomes a tool to further explore cell and tissue properties.

With new techniques available, we are increasingly able to investigate multiple levels of biological organization at the same time. For example, gene silencing via RNAi combined with live imaging of fluorescent constructs that afford cell and tissue resolution allows us to visualize the full developmental phenotype resulting from a given genetic manipulation, with *Tribolium* serving as a particularly amenable comparative model among the insects (Sarrazin et al., 2012; Benton et al., 2013; Panfilio et al., 2013; Koelzer et al., 2014). Also, as pioneered in *Drosophila*, mechanical manipulations provide a means of circumventing genetic manipulation when examining cell, tissue, and egg system levels (e.g., Ma et al., 2009; Monier et al., 2010; Wells et al., 2014), and clonal analysis approaches test cellular behaviors at tissue boundaries (Külshammer and Uhlirova, 2013). From all of these studies it becomes increasingly clear that the interplay between the levels is rather similar to a regulatory network (as known from gene interactions), including various interactions and feedback loops, than to a hierarchical structure based on increasing complexity.

Having understood the interplay of the developmental levels within a species, we can now start comparing different species and additional levels. For example, a key aspect of epithelial morphogenesis is the structure of boundaries between different tissues, where mechanical forces are transmitted and inter-tissue attachments are made. To what extent are mechanical, geometric properties of tissues and the egg system a better predictor than phylogenetic relatedness of how similar two species’ morphogenetic processes will be? Moving beyond the confines of the egg system, an even more integrated view of the phenotype can be extended to the influence of the external environment, as addressed in the growing field of eco-evo-devo (Gilbert and Epel, 2008; Abouheif et al., 2014). Ultimately, as the number of comparative animal models and accessibility of experimental tools increases, so too should the sophistication of our phenotypic understanding of how development has evolved.

## Author Contributions

KAP conceived the idea for the manuscript. KAP, TH, and MH jointly wrote the manuscript.

### Conflict of Interest Statement

The authors declare that the research was conducted in the absence of any commercial or financial relationships that could be construed as a potential conflict of interest.
